# A 3D-Printed Oxygen Control Insert for a 24-Well Plate

**DOI:** 10.1371/journal.pone.0137631

**Published:** 2015-09-11

**Authors:** Martin D. Brennan, Megan L. Rexius-Hall, David T. Eddington

**Affiliations:** Dept of Bioengineering, University of Illinois at Chicago, Chicago, Illinois, United States of America; Texas A&M University, UNITED STATES

## Abstract

3D printing has emerged as a method for directly printing complete microfluidic devices, although printing materials have been limited to oxygen-impermeable materials. We demonstrate the addition of gas permeable PDMS (Polydimethylsiloxane) membranes to 3D-printed microfluidic devices as a means to enable oxygen control cell culture studies. The incorporation of a 3D-printed device and gas-permeable membranes was demonstrated on a 24-well oxygen control device for standard multiwell plates. The direct printing allows integrated distribution channels and device geometries not possible with traditional planar lithography. With this device, four different oxygen conditions were able to be controlled, and six wells were maintained under each oxygen condition. We demonstrate enhanced transcription of the gene VEGFA (vascular endothelial growth factor A) with decreasing oxygen levels in human lung adenocarcinoma cells. This is the first 3D-printed device incorporating gas permeable membranes to facilitate oxygen control in cell culture.

## Introduction

Here we report on the development of 3D-printed microfluidic devices for the control of oxygen in cell culture microenvironments. We demonstrate a device that nests into a 24-well culture plate to control gas in each row of the plate independently of the incubator’s condition. This expands on our previous work of a device fabricated using PDMS molds and planar lithography for 6-well plates [1, 2]. The ability to independently control oxygen across each row of the plate enables more efficient experiments as a separate incubator or hypoxic chamber is not needed for each condition.

3D printing of microfluidic devices enables rapid, one-step fabrication of complex designs infeasible to make with planar lithography and replica molding techniques [3, 4]. In addition, planar lithography is time consuming, requires specialized equipment and facilities, and has a high failure rate. It is not unusual for microfluidic labs to make ten microfluidic devices to guarantee one will work properly. On the other hand, 3D CAD printing allows for unambiguous specifications and nearly eliminates time and effort spent on fabrication which may be outsourced to a 3D printing company for around $200/device [5]. 3D printing also allows integration of complex geometries not possible with planar lithography, such as hose barbs and luer fittings. Dissemination and distributed production is also vastly simplified due to easy sharing of the design as a CAD file. Due to these inherent advantages 3D printing has emerged as a method for directly printing complete microfluidic devices [5–9]. Many prototypical microfluidic device features have been recreated with 3D printing as a proof of concept for this new fabrication technique [5, 8] including modular re-configurable units [9–11]. 3D printed devices have been used for neuroengineering applications [12], inexpensive and high-throughput reactionware, [13–16], culturing and imaging arrays of seedlings [17], measuring dopamine and ATP levels in biological samples with an integrated electrode [18], or plate reader [7], and a bacteria separation flow assay [19, 20]. Other 3D-printed fluidic devices include pneumatic valves [21] a custom NMR cell [22], a rapid reconstitution package for lyophilized drugs [23] and flow plates for a water electrolysis system [24].

Printing is currently limited in choice of substrate compatible with the 3D printing process. Substrate options include many proprietary formulations which have been successfully used in a variety of applications. New techniques for using 3D-printed molds to produce devices [25–29] are also being developed, including fugitive ink methods [30–32]. To date, there are no widely available methods or materials to facilitate direct printing of gas-permeable materials, although this area is actively being explored [33]. Microfluidic cell culture devices are most commonly cast in PDMS as it is a convenient material for cell studies due to its biocompatibility, optical properties, and gas permeability, facilitating oxygen control of cell environments [34, 35]. In this study, a larger 24 well version was developed and optimized which includes several key improvements over the previous 6-well version.

Oxygen control in cells studies is often overlooked by researchers, but important for mimicking conditions experienced by cells in vivo. Typically cell culture studies are performed at 21% oxygen, atmospheric oxygen conditions, although levels that cells experience in vivo are less than 21% [35]. For example, tumors are generally hypoxic as cancer cells rapidly outgrow their vasculature creating a poorly perfused, hypoxic inner region [36]. Studying cancer cells under controlled hypoxic conditions is important in understanding the pathophysiology because research has shown hypoxia may enhance aggressive phenotypes, tumor progression, metastasis, and resistance to therapy [37–39]. Hypoxia is known to alter the transcription of many genes which are under the activity of the HIF (hypoxia inducible factor) family of transcriptional factors [40–42]. To better study the role of oxygen levels in cancer gene expression, a gas controlled culture system is required.

Previously, we developed multiwell inserts for 6-well plates that controlled oxygen in a standard off-the shelf well plate [1, 2]. These 6-well devices were completely cast from PDMS and fabricated in a multiple bonding procedure. In addition, tubing was used to connect each pillar which became quite cumbersome when moving to a newer 24-well version. Utilizing 3D printing for the fabrication allowed further features to be incorporated such as integrated distribution channels to eliminate the connection tubings between wells and hose barbs for better tubing connections, while eliminating fabrication time and failure rate. Although available 3D printable materials are gas-impermeable, the addition of a PDMS membrane following printing of the passive microfluidic network is a simple addition allowing gas transfer. In this case, having a gas-impermeable material for the bulk is advantageous as it reduces unwanted gas transfer from the bulk material prior to the gas exchange interface. The previous PDMS devices were Parylene coated along the convection channels to eliminate exchange of gas from the PDMS bulk, which again added to their complexity and is no longer needed. Convection and distribution of the gas is done in the impermeable material, eliminating dilution of the gas before reaching the diffusion layer.

## Materials and Methods

### Design of insert

**Fig 1:**
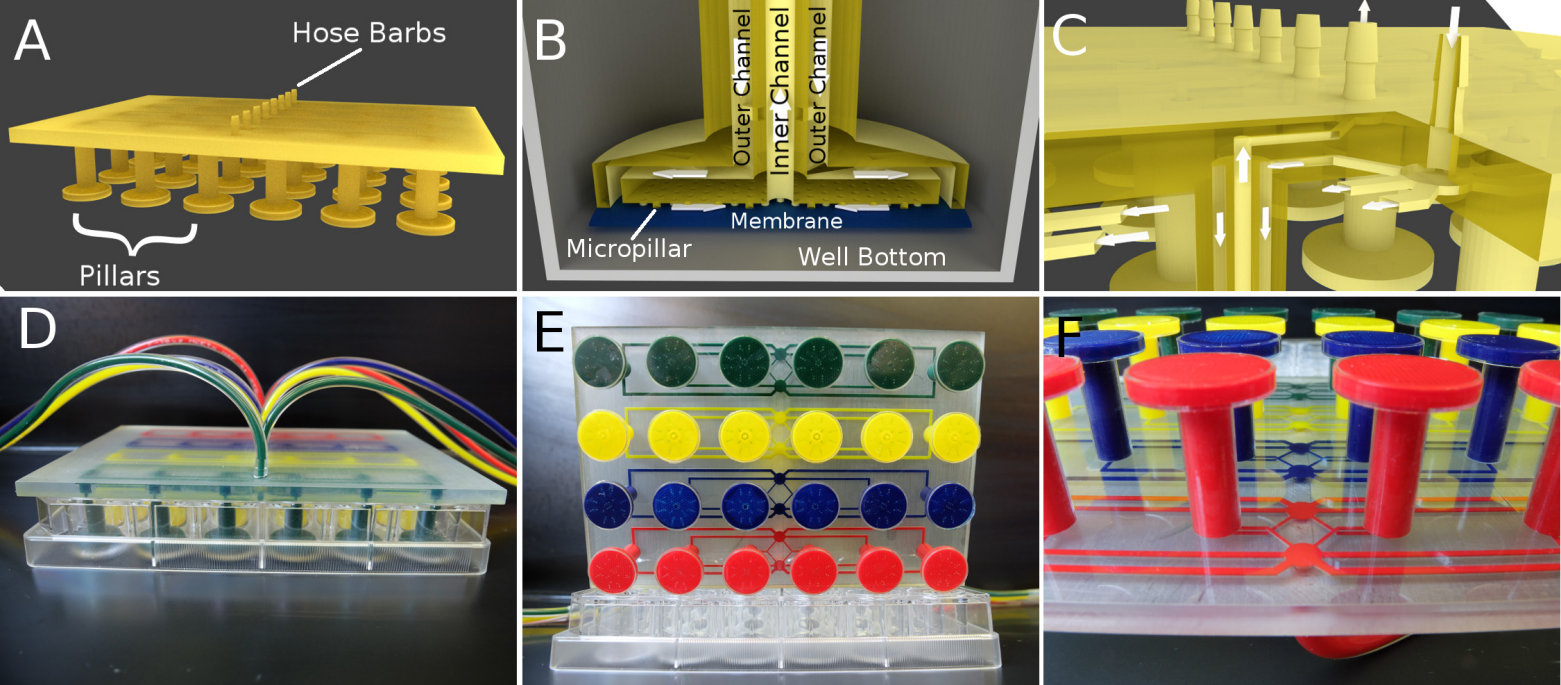
Design of 24-well Insert Device. (A) Rendering of whole 3D printed part. An inlet and outlet barb allows perfusion of gas to control 6 wells. (B) At the bottom of each pillar gas entering from the outer channel flows along the PDMS membrane (blue), which is supported by micropillars, and exhausts via the inner pipe. Diffusion occurs rapidly through the PDMS membrane to the cell culture spaced 500 *μ*m away at the bottom of the well. (C) Cross-section demonstrating how the microfluidic distribution network and double pipes are connected. The two adjacent mirrored distribution networks are spaced 1 mm apart along the z-axis allowing them to overlap and enter the separate vertical pipes. The arrows indicate the flow direction. The incoming gas enters the outer pipe on its way to the bottom of the well and returns through the inner pipe. (D) Photo of the device with dyed channels in a 24-well plate. (E) Photo of the device from the bottom with four independent channel networks. (F) Photo of the printed distribution networks.

The device was designed to integrate with a multiwell format, specifically an off-the-shelf 24-well plate. The 24-well plate insert was designed to control gas in 4 rows of 6 wells each. Each of the 4 rows can be controlled independently from an input and also incorporates an integrated distribution network and hose barbs to simplify device operation. The pillars extend into each well leaving a ∼ 500 *μ*m gap between the diffusion membrane and the culture surface. This gap allows space for ∼ 0.17 mL of media. Diffusion occurs rapidly across this gap allowing control of the dissolved gas environment around the cells. A distribution network stems from the central input that equalizes the flow along each path length by varying the channel width to the proximal, intermediate, and distal wells (Fig 1). The device also features a pipe within a pipe design so that gas flow enters and leaves the diffusion area in a uniform, and symmetrical flow pattern, which would not be possible with standard lithography and demonstrates the capabilities of 3D printing (Fig 1).

### Fabrication of the insert

CAD models were designed in Autodesk and Blender and printed via stereolithography in Watershed XC by Fineline Prototyping. The 3D part contained the microfluidic delivery channels, and was completed by adhering a gas-permeable membrane of PDMS to enable diffusion of gas to the culture area. The PDMS membranes were fabricated by spin coating 10:1 PDMS (Sylgard 184) on a silicon wafer at 900 RPM to a thickness of ∼ 100 *μ*m and curing at 70°C for 30 minutes. The PDMS membrane was cut to size and attached to the 3D-printed network with an additional, thin layer of PDMS that was applied as an adhesive to the membrane, secured in position on the end of each pillar, and allowed to cure in place.

### Oxygen characterization

Oxygen was measured with PtOEPK (Pt(II) Octaethylporphine ketone) planar sensors that were fixed to the bottom of a 24-well plate. Sensors were prepared as discussed previously [43, 44]. Briefly, polystyrene was dissolved in toluene to form a solution to which PtOEPK (Frontier Scientific) was mixed in before spin coating to a thin layer and allowing the toluene to evaporate. Five calibration points were fitted to a two-site model [45, 46] of the Stern-Volmer relationship which was used to correlate sensor intensities with oxygen concentration.

Gas was pulled through the device with negative pressure as a precaution to avoid bulging or detaching membranes, as well as bubble formation in the culture media. The outlet ports were connected to a water aspirator which pulled gas through the device from the inlet ports via a flow meter (FL-5311G; Omega) set at 50 ccm. The inlet tubing was placed in an open cone that was flooded with gas from a tank with a flow rate of 30 ccm which exceed the vacuum flow rate (12.5 ccm per 6-well network) so that the gas of interest was pulled into the inlet rather than room air due to the open connection. In this way, the pressure, and therefore flow rate, was equal across the 4 networks without having to balance flow from 4 pressurized sources, yet different gases of interest can be perfused via vacuum.

Initially the inlets are fed with a 5% CO_2_, balanced air tank until the intensity stabilized. The inlets were then switched to the control gas of either 0, 5, 10, or 21% O_2_, each with 5% CO_2_ and balanced with N_2_. Intensity measurements were then taken every five minutes for six hours. All gas tanks used in the oxygen characterization were 5% CO_2_ in addition to the desired gas mix in anticipation of cell culture experiments (where CO_2_ is necessary to buffer the culture media) as CO_2_ can alter the fluorescence of the PtOEPK. The this entire procedure was repeated three independent times.

### Cell culture

The assembled device was sterilized by spraying with 70% ethanol solution and placed in a biosaftey hood with UV lights overnight. Human lung adenocarcinoma (A549) cells were seeded at 30,000 cells per well in a 24-well plate. Cells were cultured in DMEM supplemented with 10% FBS. After reaching ∼ 70% confluency, the 24-well insert was placed in the plate and the gases were perfused through the device in the same scheme as in the oxygen characterization. Each row of six wells experienced either 0%, 5%, 10% or 21% O_2_, with 5% CO_2_ and balanced nitrogen. The assembly was placed in an incubator at 37°C for six hours.

### Quantitative PCR

Following six hours of oxygen modulation, the 24-well oxygen control insert was removed and culture media was aspirated. No contamination of the culture was observed. Cells in each well were washed twice with PBS, and 300 *μ*L of a lysis solution consisting of 10 *μ*L of *β*-mercaptoethanol (Sigma Aldrich) per 1 mL of lysis buffer (PureLink RNA Mini Kit; Invitrogen) was added to each well. The lysate from each well was collected as a sample. RNA was extracted according to the PureLink RNA Mini Kit manufacturer’s instructions.

Synthesis of cDNA was performed using the High-Capacity cDNA Reverse Transcription Kit (Applied Biosystems), and quantitative PCR was carried out on ABI PRISM 700O (Applied Biosystems) in 25 *μ*L reactions using Taqman Gene Expression Assays. Beta-2-microglobulin (B2M) served as the endogenous control for calculations of relative gene expression. Six technical replicates from the 6-well control unit were processed for each of four oxygen conditions (0%, 5%, 10% and 21% O_2_) in three independent experiments.

### Statistical analysis

Cell culture experiments were repeated three independent times. Data are expressed as the mean ± SD. One-way ANOVA with Tukey’s multiple comparison post-test performed by Prism 5 (Graphpad) determined significance.

## Results

### Oxygen control

**Fig 2:**
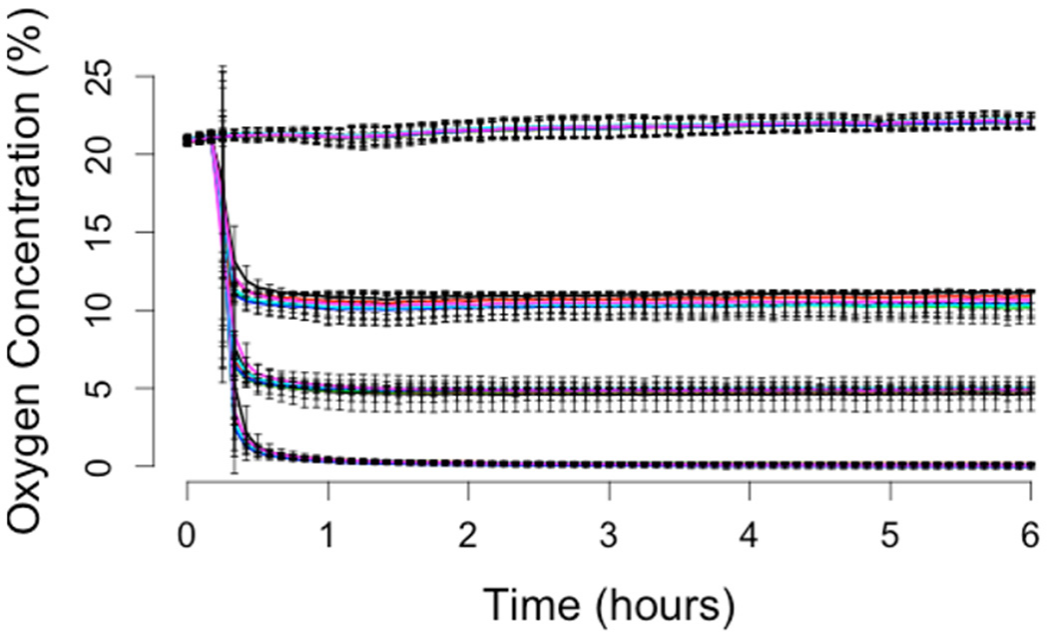
Oxygen Characterization. Time course data of four oxygen conditions are demonstrated in a 24-well plate. Each 6-well row of the plate can be controlled independently. Error bars are the standard deviation N = 3.

Oxygen control in the 24-well insert device was quantified with a platinum-based (PtOEPK) planar oxygen sensor placed at the bottom of the well, as shown in Fig 2. The device reaches steady state in 30 minutes and can then hold the oxygen level near 0% O_2_ indefinitely, while 95% N_2_, 5% CO_2_ is pumped through the device. A different gas condition can be used in each separate iteration of the 6-well unit allowing 6 technical replicates of up to 4 different gas conditions in one 24-well plate. There was little variation in the oxygen levels measured in each of the 6 wells controlled by a single inlet, demonstrating the distribution network worked effectively.

### Bioverifcation

**Fig 3:**
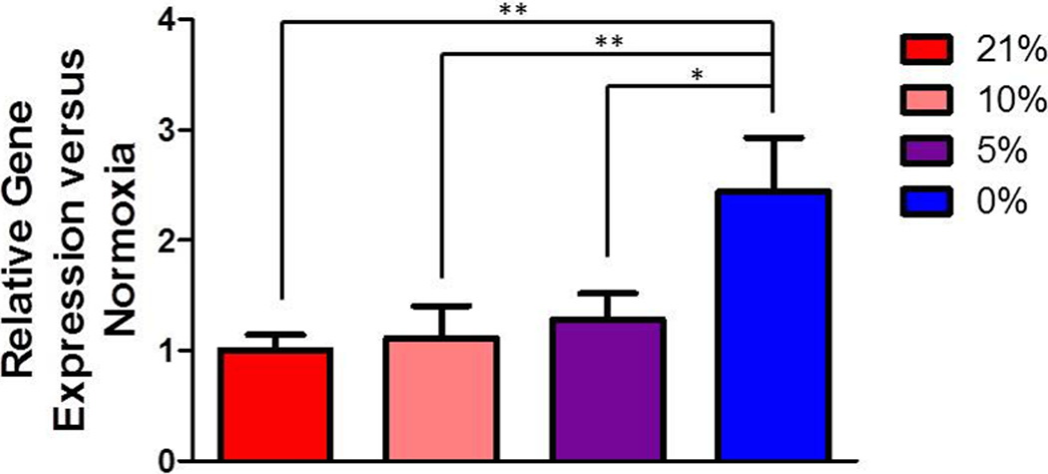
PCR Data. VEGFA expression in A549 cells after exposure to different oxygen conditions set-up by the insert device. Data are expressed as mean ± SD, N = 3.

Oxygen levels of 21%, 10%, 5%, and 0% were applied to each set of 6 wells in which A549 cells were cultured. Measurement of relative gene expression confirmed biological responsiveness of cells under the device’s control. The device’s ability to control the gas environment was demonstrated with hypoxia-induced upregulation of VEGFA mRNA in the A549 cells (Fig 3). Quantitative PCR showed a significant increase in the relative gene expression of VEGFA in wells exposed to 0% O_2_ for 6 hours as compared to 21% O_2_. Hypoxia-induced alterations in gene expression usually indicate a HIF-transduced hypoxic signal in cells. When a HIF transcription factor undergoes nuclear translocation, it can initiate transcription of genes by binding to the hypoxia response element (HRE) [47] on its target genes. VEGFA has previously been shown to have a functional HRE, and upregulation has been observed in A549 cells under hypoxic conditions [48]. The 6 h time point was chosen based on the HIF-1*α* timecourse in A549 cells. HIF-1*α* protein levels are maximal at 4 h and steeply decrease by 16 h [49]. Based on the known time course, we looked at a time point slightly delayed from the 4 h peak to account for transcription of the downstream target genes.

### Discussion

This new iteration of the multi-well insert for oxygen control maintains the convenient features of the previous design while improving distribution and interfacing with tubing due to the 3D-printed element. Oxygen control is performed in an off-the-shelf culture plate allowing standard protocols to be used for analysis assays or imaging where additional protocols are often required when cells are seeded within microfluidic chips. The VEGFA gene expression in A549 cells confirms a biological response using the multi-well insert.

This 3D-printed device improves on our previous design in a number of ways. First, the fabrication is greatly simplified. No microfabrication, PDMS molding or Parylene coating is required. Additionally, the non-permanent adhesion of membranes to the 3D-part allow them to be removed and replaced if they are damaged. In PDMS devices the layers are bonded permanently via plasma bonding so damage to a membrane is not repairable. 3D-printing also enables higher levels of intricacy without any additional fabrication, allowing us to include the distribution network, overlapping of channels in the z-axis, and the ‘pipe within a pipe’ design. A comparable PDMS device would require several additional microfabricated layers to be manually aligned to reproduce just the distribution network.

Moving forward with additive manufacturing of microfluidic devices is desirable as it will enable highly integrated designs without any additional fabrication. For this device an in-device gas mixer could allow more conditions in a single plate, perhaps even one per well or even gradients within wells while reducing the number of gas tanks required as inputs. This design could also be applied to higher format plates such as 96-well plates.

## Conclusion

3D printing microfluidic chips have been limited to oxygen-impermeable materials. We demonstrate oxygen control in 3D-printed devices with the addition of a gas permeable PDMS membrane. Oxygen control is demonstrated in a 24-well plate and PCR detection of upregulation of VEGFA mRNA in A549 cells shows the device effectively controls oxygen as expected. 3D printing allows complex designs, integrated tubing connectors, and is comparable in price to standard PDMS fabrication. This technique represents a bridge to commercialization where robust devices can be more easily shared and disseminated. While injection molding, hot embossing, or other industrial processes are cheaper when making hundreds to thousands of devices, it is not practical to make an injection mold when making tens to hundreds of devices. In addition, PDMS fabrication would be too time consuming, expensive, and the failure rate would be unacceptable. 3D printing is an excellent solution to these device fabrication needs.

## Supporting Information

S1 Dataset Oxygen Characterization Dataset. This dataset consists of intensity values of the oxygen sensors as well as the analyzed oxygen values.
(ZIP)

S2 Dataset VEGF Dataset. This dataset consists of quantitative real-time PCR for genes VEGFA and B2M.
(XLSX)
